# Nanofeatured surfaces in dental implants: contemporary insights and impending challenges

**DOI:** 10.1186/s40729-024-00550-1

**Published:** 2024-07-04

**Authors:** Keiji Komatsu, Takanori Matsuura, James Cheng, Daisuke Kido, Wonhee Park, Takahiro Ogawa

**Affiliations:** 1 Weintraub Center for Reconstructive Biotechnology, UCLA School of Dentistry, Los Angeles, USA; 2 Division of Regenerative and Reconstructive Sciences, UCLA School of Dentistry, Los Angeles, USA; 3 Section of Periodontics, UCLA School of Dentistry, Los Angeles, USA; 4 Department of Dentistry, College of Medicine, Hanyang University, Seoul, Korea; 5 Weintraub Center for Reconstructive Biotechnology, Division of Regenerative and Reconstructive Sciences, UCLA School of Dentistry, 10833 Le Conte Avenue B3-087, Box951668, Los Angeles, CA 90095-1668 USA

**Keywords:** Dental and orthopedic implants, Osseointegration, Bone-titanium integration, Osteoblasts, Microrough surface

## Abstract

Dental implant therapy, established as standard-of-care nearly three decades ago with the advent of microrough titanium surfaces, revolutionized clinical outcomes through enhanced osseointegration. However, despite this pivotal advancement, challenges persist, including prolonged healing times, restricted clinical indications, plateauing success rates, and a notable incidence of peri-implantitis. This review explores the biological merits and constraints of microrough surfaces and evaluates the current landscape of nanofeatured dental implant surfaces, aiming to illuminate strategies for addressing existing impediments in implant therapy. Currently available nanofeatured dental implants incorporated nano-structures onto their predecessor microrough surfaces. While nanofeature integration into microrough surfaces demonstrates potential for enhancing early-stage osseointegration, it falls short of surpassing its predecessors in terms of osseointegration capacity. This discrepancy may be attributed, in part, to the inherent “dichotomy kinetics” of osteoblasts, wherein increased surface roughness by nanofeatures enhances osteoblast differentiation but concomitantly impedes cell attachment and proliferation. We also showcase a controllable, hybrid micro-nano titanium model surface and contrast it with commercially-available nanofeatured surfaces. Unlike the commercial nanofeatured surfaces, the controllable micro-nano hybrid surface exhibits superior potential for enhancing both cell differentiation and proliferation. Hence, present nanofeatured dental implants represent an evolutionary step from conventional microrough implants, yet they presently lack transformative capacity to surmount existing limitations. Further research and development endeavors are imperative to devise optimized surfaces rooted in fundamental science, thereby propelling technological progress in the field.

## Introduction

For numerous years, titanium implants have served as the gold standard intra-osseous anchor in dental and orthopedic interventions. In 1952, Brånemark made the groundbreaking discovery that mechanically polished pure titanium could directly integrate with bone, leading to osseointegration—characterized by the formation of bony tissue around the implant without the growth of fibrous tissue at the bone-implant interface [1]. This pivotal concept of osseointegration continues to underpin current implant technologies and drives the exploration of novel materials and surfaces for diverse medical applications.

Osseointegration is initiated by the adsorption of proteins and cells to titanium surfaces [2–9]. Undifferentiated bone marrow-derived mesenchymal cells attach and settle on titanium surfaces to achieve direct titanium-bone contact by proliferating and differentiating to form bone tissue on the titanium surface [10–16]. Osteoblast behavior - an important factor for successful osseointegration - is influenced by the presence of titanium [11, 12, 17], and topographical [14, 15, 18–26], chemical [19, 27, 28], and physicochemical [29–33] attributes of the titanium surface. Of these three factors, surface topography has been the primary focus for improving osseointegration, and surface modifications of titanium have had significant scientific and commercial impact [34–43]. In the 1980s, attempts were made to create rough titanium surfaces by using titanium plasma-spray (TPS) [44, 45] and hydroxyapatite (HA) coatings [46–48], which showed a relatively high degree of roughness with irregularities and structures 6–10 μm wide and high [49]. Although animal experiments with these surfaces were promising, this surface texturing accelerated deposition of biofilm and tartar [50], and dissociated or solubilized coating resulted in cytotoxicity and inflammation [51, 52]. Introduced as a new modality in the 1990s, acid etching creates a rough titanium surface [53–56] of 1–5 μm pits or compartmental structures with excellent in vitro [10, 14, 18, 23, 24, 57–63], in vivo [15, 28, 57, 64–67], and clinical outcomes [68, 69]. Although other surface features such as oxidized [26, 70, 71], sandblasted [72, 73], and alkaline-treated [22, 74] surfaces can be considered microrough, acid-etched titanium surfaces have become the commercial standard for dental implants.

**Fig. 1:**
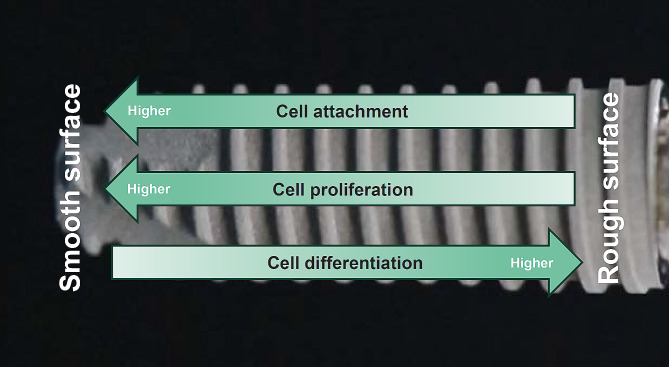
Osteoblast behavior on implant surfaces. The frustration of implant surfaces arises from the inherent behavior of osteoblasts (see Fig. 3). There is a dichotomy in osteoblastic behavior: high levels of proliferation and differentiation cannot be achieved simultaneously. Osteoblasts exhibit robust differentiation on currently used microrough titanium surfaces, while their proliferation is significantly reduced. Additionally, the number of osteoblasts attaching to microrough surfaces is compromised compared to machined, smooth surfaces

**Fig. 2:**
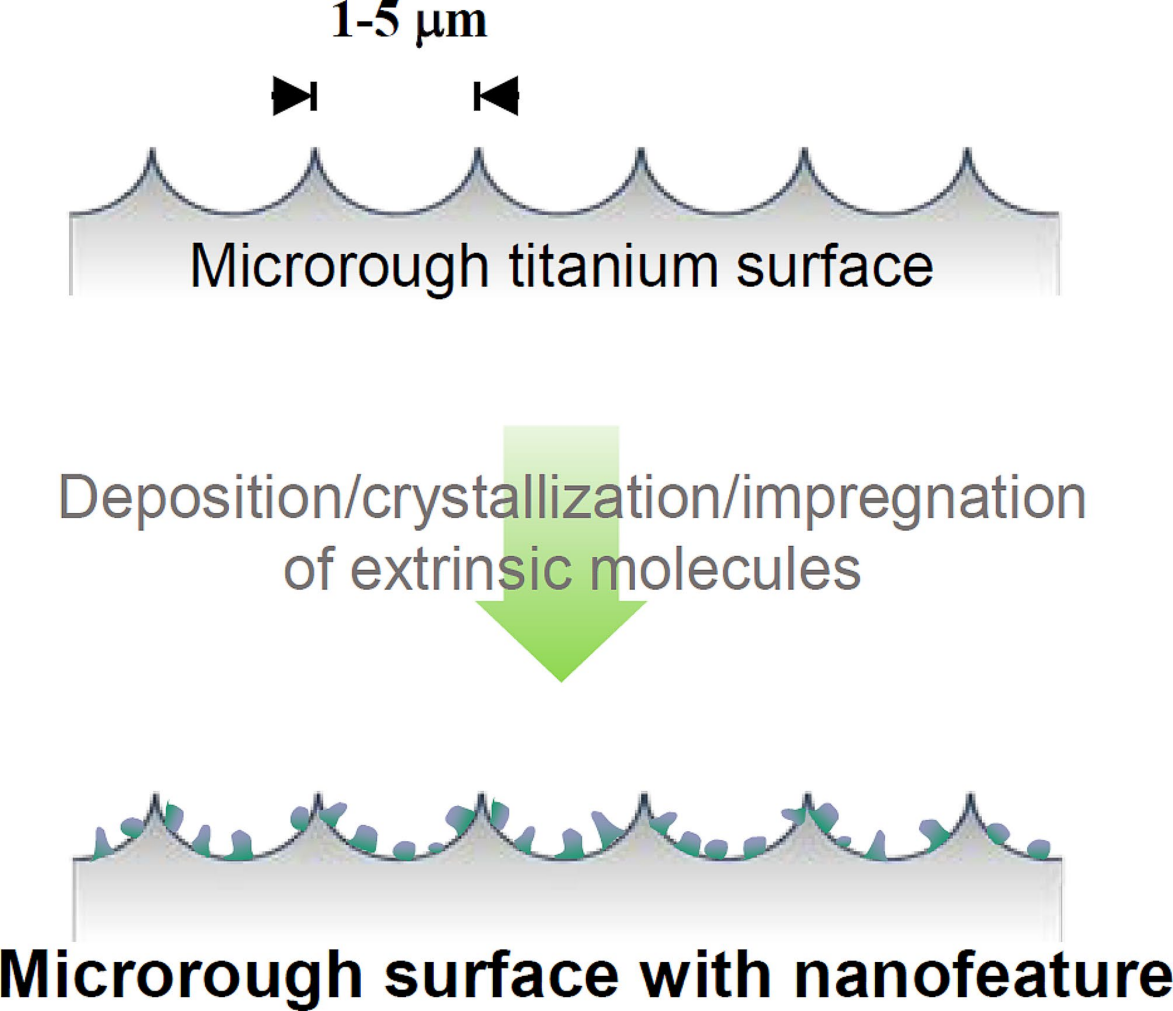
A fundamental strategy employed to develop nanofeatured surfaces in dental implants involves adding nano-scale structures, less than 100 nm in size, to existing microrough surfaces via chemical methods. This approach aims to introduce new functions, mitigate existing disadvantages, while preserving the advantages of microrough surfaces. Importantly, the added nanofeatures are not crafted with titanium oxide but rather with extrinsic molecules

Despite notable advancements in surface technology, microrough surfaces continue to present clinical challenges, including: (1) extended healing periods required for achieving osseointegration [75–78]; (2) restricted indications for implant therapy attributed to local and systemic host factors [79–87]; and (3) a success rate that has plateaued at approximately 92% [83, 88–91]. These challenges are partially attributable to a suboptimal bone-implant contact (BIC) ranging from 47.8 to 75% for commonly utilized modern titanium implants [92–99], significantly below the ideal 100%. This stagnation is closely linked to the behavior of osteoblasts on rough surfaces; while increased surface roughness enhances osteoblastic differentiation, it simultaneously inhibits proliferation [13, 54, 60, 100–106], as illustrated in Fig. 1. In response, nano-scale surface texturing for dental implants has emerged with the expectation of addressing these clinical and biological hurdles. A fundamental strategy has been to incorporate nanofeatures onto existing microrough surfaces using various chemical deposition or alteration methods with the intention of introducing new functions and mitigating disadvantages while preserving existing advantages (Fig. 2). Although commercial products such as SLActive® (Straumann®), OsseoSpeed (Astra®), and NanoTite™ (Zimmer Biomet) with nanofeatured surfaces are clinically available, a comprehensive review encompassing their morphology, physicochemical properties, biological characteristics, and the interplay between morphology and biology is lacking. Therefore, this review aims to: (1) elucidate the advantages and limitations of current microrough implant surfaces concerning their osseointegration potential; (2) evaluate the potential impact of nanofeatured titanium surfaces by employing controlled hybrid micro-nano-textured models; and (3) discuss the existing morphological and biological features of commercial nano-textured dental implants.

**Fig. 3:**
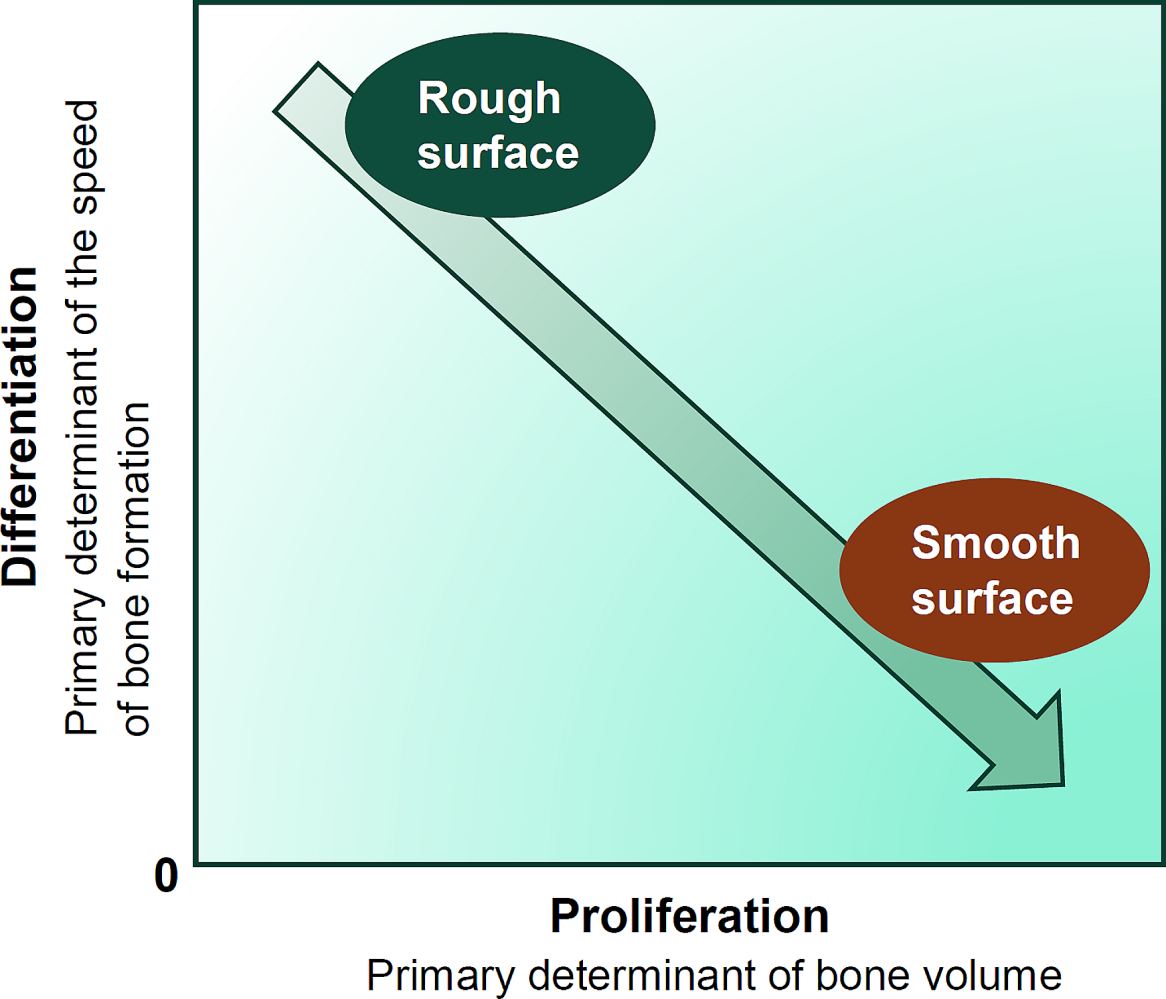
The dilemma of implant surfaces arises from the inherent dichotomy of osteoblast behavior, characterized by an inverse correlation between proliferation and differentiation. Current dental implant surfaces, typically featuring microrough textures, prioritize promoting differentiation while compromising proliferation. Essentially, this means that while bone formation occurs more rapidly on microrough surfaces, the overall bone volume tends to be reduced

## Microrough surfaces and osseointegration

Microroughened titanium surfaces were introduced to the dental implant market in the mid-1990s. The term “microrough surface” serves as a broad classification encompassing various approaches and surface topographies that induce surface roughness and irregularities at the microscale level [41, 107]. Indeed, the morphology and texture of microrough surfaces exhibit significant variability [15, 18, 63, 107]. As the most commonly used microrough titanium surface in dental implants, we first discuss primarily acid-etched microrough surfaces, which may or may not incorporate sandblasting as a pre-roughening step to create distinct levels of microroughness.

### Recruitment and attachment of osteogenic cells

The initial step crucial for osseointegration is the recruitment of osteogenic cells. Upon implant introduction into the body, circulating adhesion proteins swiftly adsorb onto the titanium surface [108], providing a scaffold and bridge for circulating undifferentiated bone marrow-derived mesenchymal stem cells (hMSCs) to settle and proliferate [109]. With subsequent cycles of proliferation, these stem cells differentiate into osteoblasts on the titanium implant surface [110].

Assessing the ability of implant surfaces to recruit and facilitate the attachment of osteogenic cells can be achieved by quantifying the number of cells attaching during the initial stage of cell culture, particularly within the first day or two before proliferation commences. Despite the general assumption that microrough surfaces promote bone-titanium integration in vivo, it’s noteworthy that microrough surfaces actually permit lower levels of osteoblast attachment compared to mechanically machined, turned, or polished (i.e., smooth) titanium surfaces [58, 60, 103]. An in vitro study demonstrated that the number of primary cells adhering to microrough surfaces is approximately half to one-fourth of those adhering to machined surfaces during the initial culture period [60, 103]. Additionally, a study utilizing MG63 osteoblast-like osteosarcoma cells reported that after 24 h of culture, approximately four times more cells adhered to smooth surfaces than to microrough surfaces [111].

While several studies have suggested that smooth titanium surfaces promote greater cell attachment compared to rough surfaces, a few studies have indicated no significant differences between rough and smooth surfaces [112], or even greater attachment on rough surfaces [113]. These conflicting results may stem from technical factors such as the method of rough surface creation, the different methods used to assess cellular attachment [114], or variations in experimental conditions [103]. Overall, cell recruitment and attachment to titanium are compromised on well-defined, typical microrough surfaces compared to machined or smoother surfaces.

### Cell proliferation

Following cell attachment to the implant surface, their subsequent growth and proliferation significantly influence their volume and the quantity of extracellular matrix produced at the implant surface [9]. Consequently, the ability to proliferate on the implant surface becomes a crucial determinant in bone mass around the implant. Given the diminished cell attachment on microrough surfaces, it prompts the question of whether the number of cells proliferating on microrough surfaces matches those on machined surfaces.

As assessed by BrdU incorporation during DNA synthesis, machined surfaces demonstrate approximately twice the rate of cell proliferation compared to acid-etched microrough surfaces, both at day two of culture and over an extended period [115]. Furthermore, proliferation on machined surfaces consistently surpasses that on acid-etched surfaces over time, with a 40% higher rate on machined surfaces, even after seven days of culture [116]. Another study comparing machined and acid-etched surfaces unveiled a three-fold increase in cell attachment and a five-fold increase in cell proliferation on machined surfaces [58].

The impact of surface roughness on cell proliferation was further investigated across three different titanium surfaces: electropolished, etched + sandblasted, and TPS [117]. Results indicated that the roughest TPS surface exhibited approximately one-third of the cell proliferation observed on the smoothest electropolished surface at 24 h and about half at 48 h. Additionally, the result of cell doubling time supported the finding [118]. Hence, it is reasonable to conclude that the prevailing notion in implantology, where proliferation is reduced on roughened surfaces, holds true [114]. Fewer osteogenic cells are available to participate in bone formation on microrough surfaces compared to smooth surfaces.

### Osteogenic cell differentiation

Osseointegration entails the filling of the gap between the implant and bone with bone matrix and/or the initiation of bone formation occurs at the implant surface (de novo osteogenesis). The production and mineralization of bone matrix rely on osteoblast function. Thus, the differentiation of undifferentiated mesenchymal stem cells into osteoblasts constitutes a pivotal event for osseointegration. Investigating cellular differentiation primarily involves assessing specific differentiation markers (genes or proteins) expressed at different stages of differentiation. For instance, early osteoblast differentiation is characterized by alkaline phosphatase (ALP) activity and collagen type I expression, while the mid to late stages are marked by bone sialoprotein, osteopontin, and osteocalcin [65, 119].

A study exploring the impact of surface roughness on cellular differentiation using machined surfaces and surfaces sandblasted to varying degrees revealed that sandblasted titanium surfaces exhibited a significant increase in osteocalcin expression upon vitamin D3 (1,25(OH)2D3) stimulation compared to smooth surfaces [120]. Similarly, cells cultured on sandpaper-roughened titanium surfaces displayed significantly higher ALP activity than those on non-roughened surfaces [121]. Moreover, grit-blasted titanium alloy induced a notable increase in osteocalcin, transforming growth factor-β1 (TGF-β), and osteoprotegerin expression by cultured cells compared to machined alloy surfaces [122]. Numerous reports have documented enhanced cellular differentiation on rough titanium surfaces [106, 117, 123–126]. These studies unequivocally demonstrate a “dichotomy,” “dilemma,” or “trade-off” [13, 106, 127, 128] in osteoblast kinetics, where surface roughness decreases proliferation while promoting differentiation. This inverse relationship between osteoblast proliferation and differentiation is illustrated in Fig. 2, with the relevance to the roughness level of implant surfaces. This osteoblast behavior also recognized in general bone biology, with molecular mechanisms to suppress cell proliferation during promoted differentiation and vice versa. Examples include proliferation control by proliferating cell nuclear antigen (PCNA) protein and cyclin D1, whose co-expression reduces osteoblastic proliferation [129, 130]. A recent study demonstrated the upregulation of these genes and proteins on microrough titanium surfaces [13]. Additionally, Runx2 expression triggers active differentiation while guiding osteoblasts to exit the proliferation phase [131–133]. It should be noted that as shown in Fig. 2, the rate of proliferation determines the volume of bone formation, while the rate of differentiation determines the speed and maturity of bone formation.

### Intrinsic quality of osseointegration and interfacial biology and physiology on microrough surfaces

BIC and the mechanical strength of osseointegration are fundamental metrics for evaluating implant success. However, relying solely on these variables often inadequately explains the underlying phenomena or mechanisms. To comprehensively investigate osseointegration, an in vitro study assessed the hardness and elastic modulus of mineralized matrix formed on machined and acid-etched microrough surfaces [17, 60, 61]. Mechanical testing utilizing nano-indentation revealed that after 28 days of culture, the mineralized matrix synthesized on microrough surfaces was 3-3.5 times harder and 2.5-3 times stiffer than that on machined surfaces [60]. These differences were as significant as those observed with cortical and trabecular bone and were correlated with increased calcification and collagen density in the mineralized matrix on microrough surfaces. Importantly, the microrough-induced enhancement in intrinsic mechanical properties was confirmed in vivo [66]. Bone integrated with microrough implants exhibited three times greater hardness than that integrated with machined implants, both at the osseointegration interface and within the peri-implant bone. The hardness of the bone associated with microrough surfaces resembled that of native cortical bone.

Another set of studies evaluated the strength of interfacial bonding between titanium and mineralized matrix [17, 61, 62, 134]. Nano-scratch testing was conducted on in vitro-mineralized matrices formed on machined and microrough titanium surfaces over 24 days of culture [62]. The critical load required to delaminate the mineralized matrix was 70% greater for microrough surfaces than for machined surfaces, a finding supported by upregulated expression of proteoglycan/glycosaminoglycan genes on microrough surfaces [62]. Notably, the addition of glycosaminoglycan-degrading enzymes into cultures significantly reduced the matrix-titanium interfacial strength [62, 135]. These studies therefore demonstrate that microrough surfaces not only enhance bone-implant contact but also improve the biomechanical quality of osseointegrated bone and promote biological adhesion at the implant interface.

### Genuine mechanical advantages of microroughened titanium at the interface

While biomechanical studies are commonly utilized to assess the strength of implantation into bone, genuine mechanical studies are scarce. One study compared five different titanium surface topographies: (1) machined, (2) sandblasted with Al_2_O_3_, (3) acid-etched, (4) sandblasted with Al_2_O_3_ and acid-etched, and (5) sandblasted with TiO_2_ and acid-etched [23]. The degree of surface roughness resulted in a four-fold difference in the interfacial shear strength between the titanium surfaces and acrylic bone cement. These findings unveiled more significant differences than expected in the mechanical interlocking capacities of the titanium surfaces with various surface morphologies. An even more crucial implication was that the anchorage of dental implants with the same BIC could vary significantly. This study also demonstrated that the interfacial area ratio (Sdr) was the most influential factor governing implant strength, contributing to 60% of the interfacial shear strength, whereas average roughness (Sa) - the most common parameter measured in the field - contributed only 12%.

### In vivo osseointegration

Microrough surfaces have frequently been reported to enhance bone-implant integration strength, or osseointegration. However, it’s crucial to acknowledge that the strength of osseointegration is a variable measured via mechanical yield tests, representing the overall anchorage of implants. Yet, this parameter may not only correlate with the degree or speed of peri-implant bone formation but also with the quality of peri-implant bone and the simple mechanical interlocking of the bone with the implant surface. For instance, implants inducing limited osteoblast differentiation and osseointegration could exhibit high osseointegration strength purely due to high levels of mechanical interlocking. Conversely, implants with high peri-implant bone volume may demonstrate low osseointegration strength due to reduced interfacial biological adhesion and poor intrinsic bone quality. Thus, a multifaceted interpretation is required to comprehend the biological and physiological capacity of implants through comprehensive analysis encompassing in vitro, in vivo, and mechanical approaches.

In a rabbit model, the torque required for removing acid-etched microrough implants was four times higher than for removing machined implants after two months of healing [136]. A subsequent study demonstrated that osseointegration was stronger for microrough implants at both the early healing timepoint of one month and the later stage of three months [53]. Studies in pigs also exhibited an increased removable torque value for microrough surfaces created by sandblasting and acid etching compared with machined surfaces throughout the measured healing period [137, 138]. Push-in biomechanical testing in rat femurs further confirmed the advantages of microrough implants, showing a two- to four-fold increase in osseointegration strength from early- to late-stage healing [64, 65]. Notably, these studies utilized cylinder implants without screw/threads, allowing for an analysis of genuine microroughness performance. Implants with sandblasted and acid-etched surfaces demonstrated higher removal torque than implants with solely acid-etched surfaces, suggesting that surface roughness influences osseointegration [139]. A rabbit study differentiating primary stability showcased that microrough implants exhibited greater strength than machined implants, irrespective of the level of primary stability [140].

A histomorphometry study comparing machined and acid-etched microrough implants in a rabbit tibia model, with implants placed with either low or high primary stability, revealed that the BIC was higher for the microrough surfaces than for the machined surfaces under both high and low primary stability conditions [140]. The average BIC after nine weeks of healing was 50.7% for the machined surface and 69.2% for the microrough surface. However, bone volume was not reported. Another study comparing machined and acid-etched microrough implants in rat femurs demonstrated that the BIC was significantly higher for the microrough implants at early and late healing timepoints, with four-fold and 2.5-fold differences at weeks 2 and 4, respectively [98]. However, bone volume was lower for microrough implants, indicating that the increased BIC around microrough surfaces was due to decreased soft tissue intervention between the bone and implant surface. Notably, the bone around microrough surfaces appeared thin, while that around machined implants was thicker [98]. This study meticulously profiled the bone volume according to the proximity to the implant surface, revealing that the bone profile rapidly and sharply increased at the microrough implant interface and gradually and moderately increased at the machined implant interface. The model was unique and reliable, considering only de novo but not innate bone as osseointegrated, using a rectangular chamber implant to allow bone ingrowth [98]. Thus, the reduce bone volume around microrough surfaces was well explained by the above-mentioned inverse correlation of osteoblastic kinetics (Figs. 1 and 2).

### Challenges and potential solutions for reduced cell recruitment, attachment, and proliferation on microrough surfaces

Given the constraints of microrough surfaces, there is a pressing need for new materials or modifications to address the underlying scientific challenges. Specifically, solutions must be sought to enhance cellular attachment and proliferation while preserving the benefits of roughened surfaces for bone integration (Figs. 1 and 2). Microrough surfaces appear to be adversely affected by several factors, including the accumulation of organic material (carbon-containing molecules) on the surface over time and reduced hydrophilicity [30, 32, 33, 141–147]. Indeed, microrough titanium surfaces exhibit hydrophobic or hydro-repellent properties [2, 7, 77, 148–158]. Mitigating and optimizing these factors could potentially increase the bioactivity of microrough surfaces and enhance osseointegration. Several methods for modifying titanium surfaces have been proposed to improve physicochemical properties, including low-temperature plasma treatment [159, 160], ultraviolet light treatment or UV photofunctionalization [3, 31, 103, 115, 147, 161–176], and hydrogen peroxide immersion [177, 178]. Topographically, increasing the surface area of implants while offsetting the disadvantages of microrough surfaces and encouraging more cells to settle may be achievable. To this end, meso-scale surface texturing - which involves aggressive acid [54] and laser etching [5, 104, 105] to create a meso-scale configuration - could prove to be an effective strategy.

## A role of nano-topography in a hybrid micro-nano titanium model

Most studies on nanofeatured surfaces in the fields of biomaterials and engineering concentrate on creating nano-topography alone without integrating topographies at other scales, such as carbon nanotubes and lithography-mediated nano-texturing. Recent advances in nanotexturing have made it possible to produce nano-structures of various shapes, sizes, and evenness/randomness. However, these advancements have scarcely been applied to metal surfaces, particularly titanium or titanium alloy, owing to technical difficulties.

### The hybrid micro-nano-rough titanium model

Leveraging the proven advantages of microrough surfaces, one strategy to further enhance implant surfaces could involve adding nano-topography to the existing microrough topography while maintaining the micro-scale pits and compartments. This approach is expected to have no adverse impact on differentiation and mechanical interlocking at the interface but to promote new positive properties through nano-topography. From a biomimetic perspective, this strategy is biologically plausible, as biological tissues exhibit hierarchical organizations of structures and components from the micro to nano scale.

**Fig. 4:**
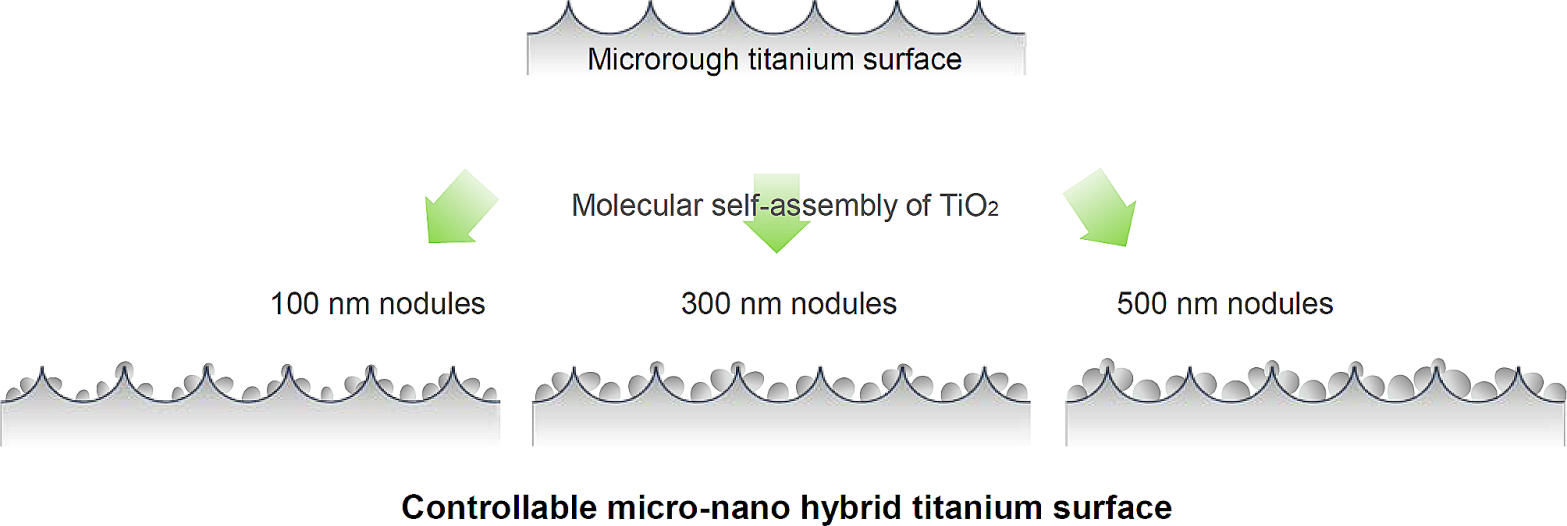
Formation of micro-nano hybrid titanium surface. TiO_2_ nano-scale nodules are genereated within micropits. This process was uncovered as TiO_2_ molecular self-assembly occurs during TiO_2_ sputter/vapor deposition onto microrough titanium surfaces. By adjusting the deposition time, the size of nanonodules can be controlled. It is important to note that the resultant surface is crafted entirely from titanium oxide, unlike the methods described in Fig. 2

**Fig. 5:**
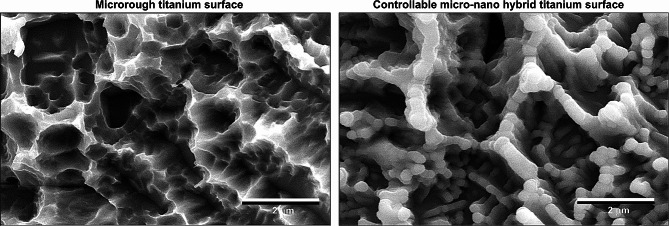
Scanning electron microscopic (SEM) images comparing the controllable micro-nano hybrid titanium surface with a typical microrough titanium surface created by acid-etching. The hybrid titanium surface was formed by allowing TiO_2_ to self-assemble on the microrough surface. The formation of 300 nm nodules at the flanks, valleys, and ridges of the micropits is visible. For detailed morphological descriptions, refer to Table 1

The discovery of molecular self-assembly of metal during sputter or vapor evaporation of metal onto pre-conditioned surfaces with specific textures has enabled the design of a titanium surface with a hybrid topography of nanonodules within micropits as illustrated in Fig. 4 [179]. This self-assembly process can be controlled to adjust the size of nanotopography, resulting in a surface that consists entirely of titanium dioxide (TiO2) without the addition of extrinsic materials. TiO_2_ was sputter coated onto acid-etched, microroughened titanium surfaces, creating nanonodules within the valleys of the micropits through molecular self-assembly [180, 181]. The resulting hybrid micro-nano surface exhibited increased surface area, robust surface roughness with geographical undercuts on the existing microrough surface, and resembled biomineralized matrices [102, 151]. Moreover, the micro-ridges/peaks were tempered. The distinctive and well-defined topography of the nanostructure, along with the significant morphological contrast with the acid-etch-created microrough surface, is depicted in the SEM image (Fig. 5) and outlined in Table 1. This self-assembly approach is versatile for creating micro-nano synergy using various combinations of targets and substrates [179]. Moreover, this approach allows the substrate to be a non-metal, such as a biodegradable polymer or collagen membrane, facilitating the production of tissue engineering scaffolds.

**Table 1:** Topographic characteristics of the micro-nano hybrid titanium surface generated through controlled TiO2 self-assembly in comparison with the acid-etch-created microrough titanium surface

Surface	Form	Ridge	Roughness	Surface area	Undercut
Microrough	Micropits	Sharp	Increased	Increased	No
Controllable micro-nano-hybrid	Nanonodules in micropits	Tempered	Further increased	Further increased	Extensive

### Biological effects of nano-topography on osteoblast function

The hybrid micro-nano titanium surfaces were optimized by controlling the size of the nanonodules through self-assembly time manipulation [180]. Osteoblasts cultured on microrough surfaces with 100-nm, 300-nm, or 500-nm nanonodules exhibited increased average roughness (Sa) from 0.35 to 1.45 mm by adding the nanonodules, while the surface area increased by 50%. The addition of nanonodules to micropits promoted osteoblast differentiation, as evidenced by the upregulation of various biomarkers [180–182]. Importantly, despite the significant increase in surface roughness, this hybrid surface enhanced osteoblast attachment and proliferation, overcoming the limitations of microrough surfaces. The increase in cell proliferation and differentiation was most pronounced when the nanonodules were 300 nm in diameter, with four- and two-fold greater cellular attachment compared to microrough surfaces at 6 and 24 h of culture, respectively [180], even surpassing that on machined, smooth titanium surfaces [102].

Biomechanical push-in testing of implants in the rat femur model demonstrated that the strength of osseointegration was over three times higher for hybrid implants with 300-nm nanonodules than for implants with microrough surfaces alone, establishing nano-in-micro titanium surfaces as a solution to mitigate the inverse relationship between osteoblast proliferation and differentiation and overcome the inherent biological challenges of rough biomaterial surfaces [180]. Interestingly, the effect of the hybrid micro-nano titanium surfaces was cell-specific, with the proliferation and function of NIH3T3 fibroblasts significantly reduced on the surface, further promoting osseointegration [102]. These distinct roles and additional advantages of the controllable micro-nano hybrid titanium surface are summarized in Table 2.

**Table 2:** Biological advantages and disadvantages of microrough surfaces and the micro-nano hybrid surface, compared to machined smooth surface

Surface	Number of cells attached	Cell retention	Cell proliferation	Cell differentiation
Microrough	Significantly less	Increased	Significantly less	Faster
Controllable micro-nano hybrid	Equivalent to smooth surface	Further increased	Not compromised	Even faster

## Current commercial nanofeatured dental implants

In the absence of nano-scale topography or structure, commercial implants, despite possessing microroughness, had often been considered to have nano-smooth surfaces [41, 42]. Consequently, in line with the strategies outlined earlier, nano-featured implants were developed by incorporating nanofeatures onto existing implants with microrough morphology. Here, we present three commercial dental implants with nanofeatured surfaces [29, 183]. In the literature, these surfaces are classified as microrough and nano-rough surfaces, as opposed to microrough and nano-smooth surfaces [41, 42].

### **Chemically-modified, sandblasted with large grit, acid-etched (SLA**®**) surfaces (SLActive**®**)**

Dental implants featuring the chemically-modified SLA® surface or SLActive® surface are crafted from Grade 4 commercially pure titanium [42] and are packaged in saline solution to mitigate exposure to air particulates, such as atmospheric hydrocarbons [184–186]. These surfaces are also proposed to foster ionic interactions between the implant surface and proteins, thereby enhancing osseointegration through the formation of nano-scale spherical saline precipitates. This mechanism, combined with its chemical modification, is presumed to be the rationale behind its designation as “chemically-modified”.

#### In vitro studies

Studies involving osteoblast-like cells cultured on machined, SLA, and SLActive surfaces indicated that cell counts on SLActive were 30% lower than on SLA after 24 h [111]. Although subsequent research comparing SLA and SLActive suggested similar levels of cell adhesion, no study has conclusively demonstrated superiority for SLActive in initial cell recruitment and attachment [187].

Regarding cell proliferation, a study compared counts of human mesenchymal stromal stem cell (hMSCs) cultured on machined, SLA, and SLActive surfaces 24 and 120 h after seeding [188]. It revealed a decrease in cell counts on rough surfaces, indicating that reduced cell proliferation was associated with enhanced cellular differentiation. Using human periodontal ligament cells, SLA surfaces exhibited greater cell proliferation than SLActive surfaces at 24 h, 5 days, and 7 days [187].

SLActive surfaces are purported to be hydrophilic in addition to their nanofeatures. However, the observed decrease in cell recruitment and proliferation on SLActive surfaces contradicts previous reports suggesting that improved hydrophilicity increases cellular attachment and proliferation [30, 33, 103, 141, 152, 181]. This discrepancy may suggest that there are only minimal superficial improvements in hydrophilicity with SLActive due to saline wetting the surface, and actual titanium hydrophilicity may not have been enhanced [189]. Rather, the results of SLActive followed the common dichotomy of osteoblasts; the addition of nanostructures by saline storage resulted in more roughness and reduced cell attachment and proliferation.

While the number of attached and proliferated cells may not increase on SLActive surfaces, studies assessing cell differentiation by measuring ALP activity and osteocalcin and osteoprotegerin expression showed significantly enhanced differentiation on SLActive compared with SLA surfaces [101, 111, 187, 190, 191]. Furthermore, SLActive surfaces have been reported to enhance the synthesis of local factors related to bone formation such as prostaglandin E2 and TGF-β and increase bone formation signaling pathway expression [111, 192, 193]. The expression of angiogenesis, fibroblast, and epithelial factors has also been reported to be higher on SLActive surfaces than SLA surfaces [194].

#### In vivo studies

A two-stage implant procedure was conducted in the maxillary anterior teeth region of miniature pigs, and BIC measurements were obtained from tissue sections at two, four, and eight weeks [184]. SLActive surfaces exhibited significantly higher BIC values than conventional SLA surfaces at two and four weeks, although this effect did not persist at eight weeks when values became similar.

Subsequent research indicated that during the initial implantation stage (approximately 2–6 weeks), SLActive yielded notably higher BIC values than SLA. However, this discrepancy diminished by the mid-term (eight weeks and beyond), with generally no significant difference between the two groups [195]. Although the final BIC values of SLActive and SLA were comparable, torque removal tests assessing the connection strength with bone suggested that SLActive exhibited greater strength at two, four, and eight weeks post-implantation. Consequently, while SLActive may not necessarily stimulate more bone formation than SLA, the mature bone formed on SLActive surfaces could contribute to the observed higher integration strength.

#### Surface characterization

Understanding the unique attributes of SLActive that contribute to its biological performance necessitates a comprehensive examination of its distinct surface characteristics. Initial studies on SLActive did not provide data or mention its nanofeatured topography. Indeed, the first reference to surface nano-topography emerged in later-stage scanning electron microscopy (SEM) analyses [29, 42, 183]. It remains unclear whether the nano-structures observed on SLActive surfaces are intentionally formed or emerge as secondary formations.

Contact angle measurements, commonly used to assess solid surface hydrophilicity, revealed intriguing differences: conventional SLA surfaces exhibited a contact angle of 138 ± 4.2°, indicating hydrophobicity or hydro-repellency, whereas the SLActive surface had a contact angle of 0°, indicating hydrophilicity or superhydrophilicity [111]. Although the manufacturer, Straumann, promotes the hydrophilic properties of SLActive, the fact that SLActive remains sealed in an ampoule filled with saline until clinical use raises questions about whether the low contact angle contributing to this hydrophilic claim might represent simply a wettability or “hydrophilic-like” phenomenon due to saline coverage.

X-ray photoelectron spectroscopy (XPS) analysis revealed a decrease in carbon content from 34.2 ± 2.0% on SLA surfaces to 14.9 ± 0.9% on SLActive surfaces [111], suggesting potential prevention of post-manufacture organic material adsorption and accumulation. Despite claims that SLActive only contacts nitrogen and saline solution after sandblasting and acid etching, the presence of carbon on the titanium surface is noteworthy, implying that carbon contamination may be ubiquitous and difficult to prevent. Another XPS analysis confirmed a reduction in carbon atoms and an increase in titanium atoms on SLActive surfaces, with a titanium content of 26.5 ± 0.9% and a carbon content of 18.4 ± 2.7%, compared to a titanium content of 18.4 ± 1.6% and a carbon content of 37.3 ± 3.4% on SLA surfaces [184]. However, the atomic percentages of sodium and chlorine, likely present from the saline, are unknown.

This paper reported no significant differences in surface roughness parameters “Sa,” “Sq,” “St,” and “Sk” between SLA and SLActive using SEM and white light confocal microscopy [184]. Similarly, another study compared surface roughness using similar methods and detected no notable differences between SLA and SLActive surfaces [111]. However, another study reported an increase from 34–97–143% in SLActive’s Sdr value, identifying nano-scale structures formed on the microrough surfaces [29]. The researchers also observed a threefold difference in the average roughness (Sa) between SLA and SLActive surfaces at the nano-scale.

Optical interferometry is significantly limited for analyzing nanoscale roughness, especially for complex objects like implants [183], due to factors such as device and lens resolution, curvature correction, area of interest, and cut-off values, which can lead to variability in Sa values. Thus, inconsistent reports of SLActive’s surface roughness might be attributed to the limitations of nanoscale topography analysis technology. Currently, direct, qualitative observation of morphology through SEM analysis might provide a more accurate reflection than quantitative analyses of surface topography. Following reports on the presence of nano-topography on SLActive surfaces using SEM [50, 64, 174], numerous spherical particle structures were discovered on this surface. However, earlier SEM analyses did not detect nanoscale structures (111), and images in studies reporting these structures depict vague, slightly granular, plate-like, or polymorphic structures (29, 42, 184, 198), making their morphologic featrue challenging to discern. The exact characteristics and origin of these nanoscale structures are not definitively identified or characterized.

A surface characterization of dried SLActive surfaces by XPS suggested that crystalline salts contribute to the nanoscale structuring, with high detected levels of sodium at 25.2% and chlorine at 16.1% [42]. However, earlier XPS analyses did not detect sodium or chlorine on SLActive surfaces, showing 100% oxygen, titanium, and carbon compositions [184]. A recent study also did not detect sodium or chlorine on SLActive surfaces [196], indicating an intricate property of the surface and a need for further research to understand the elemental composition. To the best of our knowledge, no study has definitively explored the bond strength of nanoscale structures on SLActive surfaces or the intrinsic strength of these nanostructures. Therefore, the specific contributions of these nanoscale topography/structures to the biology of osseointegration and mechanical interlocking with bone remain unknown.

### Fluoride-modified sandblasted surface (OsseoSpeed)

Astra Tech’s OsseoSpeed implants are composed of Grade 4 commercially pure titanium [42] and undergo TiO_2_ blasting followed by hydrofluoric acid (HF) treatment. HF treatment has been suggested to influence osseointegration by generating nanostructures [197–199]. This surface modification enhances bone formation and results in higher BIC within a shorter timeframe compared to TiOblast, a precursor surface that only exhibited micro-level surface roughness from sandblasting.

#### In vitro studies

The fluorine ion modification of the OsseoSpeed surface via HF treatment has been shown to promote osteoblast differentiation [200]. Studies utilizing hMSC-derived osteoblasts investigated the effects of varying titanium particle size used in sandblasting and surface fluorine concentration (%) on osteoblast function [201]. Results indicated that cell attachment before 24 h of culture did not differ significantly according to sandblasting particle size or fluorine concentration. However, cell proliferation at 24 h of culture was notably higher in the HF-treated group compared to the sandblasted group. Nevertheless, at 48 and 72 h of culture, surfaces sandblasted with 75 μm particles exhibited the highest cell proliferation, whereas HF-treated surfaces showed decreased cell proliferation as fluorine concentrations increased.

Expression levels of key differentiation genes including actin, collagen, osteocalcin, osteopontin, bone sialoprotein (BSP), and bone morphogenetic protein (BMP) were quantified using reverse transcription-polymerase chain reaction (RT-PCR) to assess osteoblast differentiation [200]. Notably, expression of osteopontin was higher on the HF-treated surface on day seven of culture compared to the untreated surface. Despite the absence of morphological differences observed by SEM surface analysis due to HF treatment, the effects of HF-treated sandblasted titanium on osteoblasts were attributed to the presence of fluorine ions rather than surface morphology. The observed negative correlation between cell proliferation and differentiation, as discussed earlier, also applies to OsseoSpeed, suggesting the necessity to address this issue. For OsseoSpeed, the presence of fluorine on the titanium surface enhanced the expression of bone formation-related genes; however, the relationship between gene expression and fluorine concentration remains unclear, and the optimal fluorine concentration for differentiation has yet to be determined. While fluorine ions have been reported to promote protein adsorption, alter adhesive proteins and subsequent cellular interactions, and stimulate localized calcium phosphate deposition, the exact mechanism of action of fluorine remains uncertain. SEM analysis of HF-treated surfaces revealed the presence of spherical structures ranging from 50 to 200 nm on the microrough surface formed by sandblasting [200].

#### In vivo studies

Titanium implant surfaces, either sandblasted or sandblasted with HF treatment, were implanted into rabbit tibiae. Subsequent histological and mechanical evaluations were conducted at one and three months of healing [202]. At the three-month mark, implants treated with HF exhibited a removal torque of 85 ± 16 Ncm, whereas those with only sandblasted surfaces showed a significantly lower removal torque of 54 ± 12 Ncm. Furthermore, the shear strength between bone and implant surface measured 23 ± 9 N/mm² for HF-treated surfaces, which was notably higher than the 15 ± 5 N/mm² recorded for sandblasted surfaces. Histological assessment indicated a higher BIC for HF-treated surfaces (35 ± 14%) compared to sandblasted surfaces (26 ± 8%) after one month of healing. This discrepancy persisted at the three-month mark, with BIC percentages of 39 ± 11% for HF-treated surfaces and 31 ± 6% for sandblasted surfaces.

A canine study revealed that OsseoSpeed implants yielded significantly greater BIC and bone fill in comparison to TiOblast implants up to six weeks post-implantation [203]. Although OsseoSpeed exhibited higher rates of new bone formation and BIC than TiOblast after two weeks, this disparity diminished by the sixth week [97]. Notably, the BIC appeared to plateau at 60% post-healing, suggesting that OsseoSpeed’s impact on final BIC strength was limited, primarily affecting the early stages of implant integration.

Comparative studies between OsseoSpeed and other implant systems have shed light on the limitations of nanotechnology. In an immediate post-extraction implantation model, no significant difference in BIC was observed between OsseoSpeed and other implant systems (Osseotite, Thommen SPI Element, and SLA surfaces) at six weeks post-implantation [204]. Similarly, a study comparing OsseoSpeed and TiUnite in rabbit tibiae found no significant difference between the two groups at the two-week mark [205]. Another study, using a rabbit tibia model, compared OsseoSpeed and sandblasted implants through pull-out tests and analysis of bone formation-related gene expression after implant removal [206]. While no significant differences were observed initially, OsseoSpeed exhibited higher pull-out strength, peri-implant cortical bone density, and expression of bone formation-related genes (osteocalcin, Runx2, and collagen type I) after eight weeks of healing. These findings suggest limited effects of OsseoSpeed during early healing stages, with more pronounced differences emerging during later stages. Supporting this, OsseoSpeed demonstrated significantly higher BICs in grafted bone and total bone (grafted + existing bone) after eight weeks of healing [207].

#### Surface characterization

The surface of OsseoSpeed, often referred to as a “fluoride-modified surface” in both scientific and commercial literature, appears to have developed its nanostructure incidentally, discovered during subsequent investigations into factors contributing to its observed biological effects. OsseoSpeed appears to have been engineered to harness the bioactive properties of fluoride ions on base grade 4 commercially pure titanium [208]. Fluoride ions are recognized for their ability to form fluorohydroxyapatite or fluorapatite, exhibiting superior crystallinity and resistance to solubility compared to hydroxyapatite within bone [209–211]. In vitro studies have demonstrated increased bone density and elevated alkaline phosphatase activity associated with fluoride ions. It has been proposed that titanium fluoride may interact with phosphate groups on the hydroxyapatite surface, potentially forming covalent bonds between titanium and bone.

Morphological evaluations of OsseoSpeed surfaces have revealed that, in comparison to untreated surfaces, the peak height (Sa) is slightly reduced, resulting in a smoother surface (Sa of 0.91 ± 0.14 μm for HF-treated surfaces versus 1.12 ± 0.24 μm for sandblasted-only implants). The Sdr values were measured at 1.21 ± 0.04% for HF-treated surfaces and 1.34 ± 0.08% for control surfaces. These assessments indicate that the surface area of HF-treated surfaces is 21% higher than that of a completely flat plane, while control surfaces increased by 34% [212]. This “smoothening” effect is thought to arise from the mild reduction in microroughness introduced by sandblasting through HF treatment. Other surface evaluations have shown a slight increase in Sa and Sdr from TiOblast to OsseoSpeed [29, 183]. Essentially, the distinguishing characteristic of OsseoSpeed can be described as the addition of visible nano-roughness [213]. However, while studies have confirmed the presence of nanostructure and fluorine modification on the OsseoSpeed surface, the data remain inconclusive regarding their individual and/or synergistic roles in specific events of osseointegration.

### Acid-etched surface with discreate calcium phosphate deposition (NanoTite™)

NanoTite™, developed by Zimmer Biomet, represents a calcium phosphate-deposited evolution of its predecessor, the Osseotite™ surface. Osseotite™ is characterized by a microrough texture composed of micron-level ridges and valleys achieved through dual acid-etching of titanium alloy using hydrochloric and sulfuric acids [214]. The final step of treatment with sulfuric acid results in a fundamental surface structure believed to share similarities with other acid-etch-created microrough surfaces from different manufacturers. It’s worth noting that Osseotite™ is crafted from grade 5 titanium alloy Ti-5Al-4 V[64], potentially introducing elemental differences in structural strength and acid resistance [215].

NanoTite™ is a depositioning/coating applied to Osseotite™ by immersing it in a diluted suspension of hydroxyapatite (HA) particles and repeating cycles of drying and immersion to create a nano-thin HA or calcium phosphate film, typically with a thickness ranging from 20 to 40 nm [216]. This immersion method for coating application is referred to as the DCD method (Discrete Crystalline Deposition of nanometer-scale CaP crystals).

The calcium phosphate deposition achieved through the DCD method is believed to promote stronger adhesion compared to the conventional plasma spraying method [217, 218]. Another distinguishing feature is that, unlike traditional HA coatings that cover the entire implant surface, the DCD method leaves approximately 50% of the surface exposed as acid-etched regions [219]. In essence, the NanoTite™ surface can be described as a hybrid surface, combining microroughness with nanostructures, effectively employing a common strategy observed in the aforementioned surfaces.

#### In vitro studies

In vitro studies concerning NanoTite are relatively limited. One study examined the impact of calcium (Ca) and phosphate (PO_4_) presence on platelet adhesion and activation. Results indicated that surfaces containing Ca and PO_4_ promoted platelet activation as surface microstructures became more intricate, as observed in SEM images [220].

Another study categorized implant surfaces into three regions - “groove,” “side,” and “top” - and assessed the characteristics of NanoTite, OsseoSpeed, TiUnite, and SLActive implants in terms of surface analysis and cell attachment [221]. Findings revealed that NanoTite exhibited the smoothest surface among the four implant surfaces across all regions of the implant threads. Cell attachment rates after 48 h of cell culture on the groove and side regions followed the order of OsseoSpeed = SLActive > NanoTite > TiUnite. SEM visualization also suggested that while cells on NanoTite grooves formed confluent layers, the thickness of the cell layer was thinner compared to OsseoSpeed and SLActive, with fewer cells attaching to the side and top regions. However, the scarcity of studies hinders a comprehensive understanding of the specific response of osteoblasts to NanoTite surfaces.

#### In vivo studies

Four types of implants were inserted into rat femurs: dual acid-etched (DAE) + HA-coated surfaces, DAE-treated surfaces, mechanically polished + HA-coated surfaces, and machined surfaces [222]. Biomechanical push-in tests were performed after a healing period of two weeks. Results indicated that the osseointegration strength for DAE + HA implants was approximately 1.3 times stronger than that for DAE implants and about eight times stronger than machined surfaces. The authors suggested that the significant improvement in surface properties was primarily due to the microroughness created by acid treatment rather than the effect of the nanostructures inherent to HA coatings. In other words, the impact of nanostructures on the implant-bone interface appears comparatively limited compared to the effects of microroughness. This hypothesis was supported by three-dimensional bone morphology measurements using µ-CT, which revealed that the volume of bone around DAE implants remained consistent regardless of the presence of HA nanoparticles. Therefore, the increased strength of osseointegration for DAE + HA implants was attributed to the enhanced interfacial adhesion strength or shear strength between the implant surface and bone, supported by previous studies indicating that implants with similar Bone-to-Implant Contact (BIC) do not necessarily exhibit the same biomechanical anchorage.

Another in vivo study compared pure titanium and titanium alloy implants in rat femurs [223]. Four implant types were used: DAE + HA-coated pure titanium (cpTi-DCD) and titanium alloy implants (Ti-6Al-4 V-DCD), pure titanium without DCD treatment (cpTi), and titanium alloy implants without DCD treatment (Ti-6Al-4 V-non-DCD). After nine days, the mechanical connection strength between bone and implants was tested. The connection strength of DCD samples (both cpTi and Ti-6Al-4 V) was significantly higher than that of non-DCD samples (both cpTi and Ti-6Al-4 V). Within the DCD group, Ti-6Al-4 V-DCD exhibited a significantly higher tensile strength of 11.3 N compared to 7.2 N for cpTi-DCD. Notably, non-DCD samples showed interfacial failure, where bone detached from the implant surface for both cpTi and Ti-6Al-4 V. Conversely, DCD samples showed no bone detachment but experienced a yield point due to cohesive failure or disruption of bone tissue. These results support the hypothesis that the HA coating induced by DCD treatment enhances the adhesive strength between bone and implant surfaces [222–224].

A study compared BICs nine days after implantation between DCD-treated and control samples [225]. DCD-treated pure titanium and titanium alloy both exhibited significantly higher BICs than the non-DCD group. Moreover, the average BICs followed the order of cpTi-DCD group > Ti-6Al-4-DCD group > Ti-6Al-4 > cpTi. Interestingly, these results contradicted earlier findings suggesting that titanium alloy is more influenced by DCD-mediated increases in adhesive strength than pure titanium.

Similar experiments were conducted in rat femurs using DAE-treated titanium (cpTi and Ti-6Al-4 V) and DAE + DCD-treated titanium implants [226]. After two weeks, there were no significant histological differences between the two groups. BIC values for DAE titanium alloy and DCD titanium alloy were 55.95 ± 11.81% and 61.10 ± 7.89%, respectively, with no statistically significant difference between groups. These results indicated that NanoTite did not have a significant positive impact on early bone formation in rats. Similar results were also reported in other studies. Titanium alloy-DCD implants showed increased BICs compared to sandblasted, acid-etched, and sandblasted + acid-etched implants in a rabbit model, although no significant differences were observed [224]. A study in dogs focusing on the comparison between Osseotite and NanoTite in the early stages of healing (week two) [227] showed no significant differences in tissue composition, including dimensions, collagen, fibroblasts, vascular structures, and white blood cells. BIC measurements for Osseotite were larger than for NanoTite, suggesting that attaching nano-sized calcium phosphate crystals to etched implants has no significant impact on initial bone formation.

Similar results were observed in an immediate post-extraction implantation model. In this study, beagle dogs were used to simulate immediate post-extraction implantation with Osseotite and NanoTite implants [228]. Although NanoTite tended to show higher values than Osseotite at weeks two and four, there were no statistically significant differences. Another dog study used SLActive and NanoTite implants and evaluated their stability and bone mass using resonance frequency analysis (RFA) and µ-CT at two, four, and eight weeks [229]. While RFA values at week eight were significantly higher for SLActive, there was no difference in bone mass between the two groups. Although this study suggests clinical superiority of SLActive over NanoTite, it is worth noting that these implants have inherently different macroscopic structures, making it difficult to directly compare their finer micro- and nano-surface structures. Indeed, there was no significant difference in BIC and bone volume between SLActive and NanoTite implants [230].

These studies highlight the need for further research to establish a consensus and understand the underlying mechanism mediating the different features of osseointegration. Specifically, it remains unclear whether this effect is due to the presence of nanostructures or the chemical action of calcium phosphate. More in vitro studies are needed to evaluate the behavior and function of osteogenic cells responding to the surfaces. In particular, the important question of whether and how the nanostructure on NanoTite contributes to the inverse correlation between osteoblast proliferation and differentiation still needs to be addressed.

#### Surface characterization

SEM and roughness analyses unveiled distinct surface morphologies for cpTi-DCD and Ti6Al4V-DCD [225]. Images of titanium alloy + DCD exhibited less defined micro-compartments with rounded tips [29, 223]. When comparing DCD-treated alloy and pure titanium, the alloy showed more CaP attachment. In essence, while microroughness decreased with DCD treatment of the alloy, it can be hypothesized that nano-roughness increases due to enhanced CaP deposition, thereby compensating for the reduction of microroughness. However, the average roughness (Sa) was lower on NanoTite than on Osseotite [50, 64], or similar between the two surfaces [226].

SEM imaging indicated significant variation in the size of calcium phosphate particles, suggesting the presence of various sizes of CaP aggregates in a random form [29, 42]. One study reported that only 1.4% of Ca was detected on the NanoTite surface, underscoring the role of low concentrations of Ca in bone formation [231]. Furthermore, clarification regarding the thickness and absorption rate of adherent calcium phosphate is deemed necessary.

Surface elemental analysis of NanoTite surfaces using XPS identified Ti at 6%, O at 53.9%, C at 18.7%, Ca at 12.3%, P at 7.9%, F at 0.5%, and S at 0.3% [42]. While Ti, O, C, Ca, and P are expected in the manufacturing process, the detection of fluorine and sulfur was unexpected and suggested residual substances from double-acid treatment or its pre-treatment.

## Discussion

Since the initial discovery that microrough titanium surfaces enhance osseointegration compared to machined smooth surfaces, many implant manufacturers have developed implants fixating with micro-texturing technique. Over time, methods for treating implant surfaces have evolved in an attempt to transition titanium surfaces from microrough to nanorough. However, currently, microrough surfaces still dominate the market, indicating that these nano-surfaces have not completely addressed the limitations seen with microrough surfaces. Indeed, some studies suggest that nanofeatured surfaces have made little difference [232], and there is a perceived lack of innovation to improve implant surfaces [233].

Therefore, a key focus of this review was to identify the challenges of successful microrough surfaces including the three commercial nanofeatured dental implants. In addition to the biological challenge of the inverse correlation between osteoblast proliferation and differentiation, we evaluated the topographical and chemical characteristics of nano-featured surfaces, as outlined in Table 3. Nano-structures on the three dental implants consisted of non-titanium oxide on titanium implants; NaCl on SLActive, titanium fluoride or fluoride impregnation on OsseoSpeed, and calcium phosphate on NanoTite, which are completely different from the controllable micro-nano hybrid TiO_2_ model described in this study. These differences may partially explain the varied osteoblast reactions observed. Biological effects of the three nanofeatured surfaces are summarized in Table 4. Osteoblasts demonstrated reduced attachment and proliferation on commercial nanofeatured surfaces, particularly on SLActive and OsseoSpeed, compared to a significant increase observed on the hybrid micro-nano surfaces. Commercial nano-implants do not appear to have a significantly increased surface area compared to the hybrid model. The size of nano-structures also appears to be crucial. Unlike the 300 nm nanonodules optimized for the most significant biological impact in the nano-micro hybrid model, the nanostructures on commercial nano-surfaces often exhibited polymorphic characteristics and very challenging identification in terms of form and size, mostly < 100 nm. Indeed, the visual representations of these three surfaces vary considerably among publications. As demonstrated in the controllable hybrid model, the 300 nm nodules were indispensable for significantly augmenting roughness and engendering undercuts, thereby contributing to the amplified surface area. In essence, the three nanofeatured surfaces did not surmount the inverse relationship between osteoblast proliferation and differentiation.

**Table 3:** Morphological and chemical attributes of commercially available nanofeatured dental implant surfaces

Surface	Base microtopography	Method to create nanofeature	Form of nanofeature	Chemistry of nanofeature
SLActive	Acid-etched, micropits	Immersion in saline	Nano-particles or polymorphic	Crystalized NaCl
OsseoSpeed	Sandblasted, micro-irregularities	Fluoric acid treatment	Nano-nodules or polymorphic	Titanium fluoride or fluorine-impregnation
NanoTite	Acid-etched, micropits	Immersion in HA suspension	Nano-particles or polymorphic	Crystalline CaP

**Table 4:** In vitro osteogenic cell response and function on commercially available nanofeatured dental implant surfaces, compared to their predecessor surfaces

Surface	Number of cells attached	Cell proliferation	Cell differentiation
SLActive	Significantly reduced	Significantly reduced	Significantly promoted
OsseoSpeed	Similar	Significantly reduced	Significantly promoted
NanoTite	Undetermined	Undetermined	Undetermined

Another identified challenge of current implants is that the final BIC does not reach 100%. Brånemark machined implants achieved a BIC of 53.9 ± 11.2% [234], and even implants treated with HF and possessing a micro-and-nano surface only achieved a BIC of 58.31 ± 5.79% [235], showing limited improvement. NanoTite surfaces show a BIC of 61.10 ± 7.89% as described earlier. SLActive implants exhibited a BIC of 82% [186], but still fall short of 100%.

Considering the identified limitations of microrough surfaces and the improvements afforded by nanofeatured surfaces, implants with nanostructures show relative improvements in osseointegration speed and early-stage BICs compared to their predecessors. However, these improvements are limited, and there is ample room for further enhancement. Furthermore, detailed studies on these nanofeatures are lacking, and the composition, mechanical properties, and specific biological effects of nanofeatured surfaces have not been fully elucidated.

## Conclusion

Implant therapy remains a cornerstone of dental treatment, yet the challenge of achieving optimal osseointegration persists. Long healing times, limited indications for implants, plateaued success rates, and a high incidence of periimplantitis underscore this challenge. Presently, commercial nano-featured implants seem to promote osteoblastic differentiation and early bone formation compared to microrough surfaces. However, they fall short of achieving near 100% bone-implant contact or significantly enhancing the final level of osseointegration attained with existing microrough surfaces. The biological hurdle of the inverse correlation between osteoblast proliferation and differentiation remains unaddressed. Nevertheless, strategies utilizing a controllable hybrid micro-nano texturing model have shown promising effects on cellular reactions, enhancing osteoblast attachment and proliferation compared to microrough surfaces while concurrently preserving or even stimulating differentiation, thereby circumventing the inverse correlation. Further research and commercial development of this promising strategy and technology platform are imperative.

## Data Availability

No datasets were generated or analysed during the current study.

## Abbreviations

ALP: Alkaline Phosphatase
BIC: Bone-Implant Contact
BMP: Bone Morphogenetic Protein
BSP: Bone Sialoprotein
DAE: Dual acid-etched
HA: Hydroxyapatite
HF: Hydrofluoric Acid
hMSCs: Human Mesenchymal Stem Cells
PCNA: Proliferating Cell Nuclear Antigen
RT-PCR: Reverse Transcription-Polymerase Chain Reaction
SEM: Scanning Electron Microscopy
TGF-β: Transforming Growth Factor-β1
TiO2: Titanium Dioxide
TPS: Titanium Plasma-Spray
XPS: X-ray Photoelectron Spectroscopy
